# Barriers Influencing Vaccine Development Timelines, Identification, Causal Analysis, and Prioritization of Key Barriers by KOLs in General and Covid-19 Vaccine R&D

**DOI:** 10.3389/fpubh.2021.612541

**Published:** 2021-04-20

**Authors:** Marga Janse, Thomas Brouwers, Eric Claassen, Peter Hermans, Linda van de Burgwal

**Affiliations:** ^1^Athena Institute, Faculty of Earth and Life Sciences, Vrije Universiteit, Amsterdam, Netherlands; ^2^Julius Centre for Health Sciences and Primary Care, University Medical Centre Utrecht (UMCU), Utrecht, Netherlands

**Keywords:** vaccine development timelines, KOLs, RCA, ranking of innovation barriers, Covid-19 vaccine development

## Abstract

A frequently mentioned factor holding back the introduction of new vaccines on the market are their prohibitively long development timelines. These hamper their potential societal benefit and impairs the ability to quickly respond to emerging new pathogens. This is especially worrisome since new pathogens are emerging at all-time high rates of over one per year, and many age-old pathogens are still not vaccine preventable.Through interviews with 20 key-opinion-leaders (KOLs), this study identified innovation barriers that increase vaccine development timelines. These innovation barriers were visualized, and their underlying causes revealed by means of qualitative root cause analysis. Based on a survey the innovation barriers were quantitatively ranked based on their relative impact on both regular, and Covid-19 vaccine development timelines. KOLs identified 20 key innovation barriers, and mapping these barriers onto the Vaccine Innovation Cycle model revealed that all phases of vaccine development were affected. Affected by most barriers is the area between the preclinical studies and the market entry. Difficult hand-off between academia and industry, lack of funding, and lack of knowledge of pathogen targets were often mentioned as causes. Quantitative survey responses from 93 KOLs showed that general vaccine development and Covid-19 vaccine development are impacted by distinct sets of innovation barriers. For the general vaccine development three barriers were perceived of the highest impact; limited ROI for vaccines addressing disease with limited market size, limited ROI for vaccines compared to non-vaccine projects, and academia not being able to progress beyond proof of principle. Of highest impact on Covid-19 vaccine development, are lack of knowledge concerning pathogen target, high risk of upscaling unlicensed vaccines, and proof of principle not meeting late-stage requirements. In conclusion, the current study demonstrates that barriers hampering timelines in vaccine development are present across the Vaccine Innovation Cycle. Prioritizing the impact of barriers in general, and in Covid-19 vaccine development, shows clear differences that can be used to inform policies to speed up development in both war and peace time.

## Introduction

No human intervention has contributed to global health more than large-scale immunization efforts by means of vaccination (1–3). Following Edward Jenner's experiments with a smallpox vaccine in 1796, the disease was eradicated in 1979 (4, 5). Other success stories include an over 95% reduction of global incidence for measles, mumps, rubella, pertussis, tetanus, and diphtheria (6). According to the estimates of the World Health Organization (WHO) immunization efforts currently prevent between 2 and 3 million deaths per year (7) and this is achieved in a remarkably cost-effective way (6, 8).

Despite this success, there is still a large unmet medical need that could be met through immunization efforts (9). For one, many diseases caused by infectious pathogens are not yet preventable by vaccines. Among these are Malaria and HIV, together causing over 175 million deaths per year (10). In addition, several pathogens for which vaccines are available still cause a large number of deaths as a result of lacking societal acceptance, prohibitive costs, or serotype mismatches (11–14). Lastly, vaccines have the potential to target a larger number of ailments and patient populations than currently is the case. Examples are cancer vaccines and vaccines targeting adults and elderly (15).

One of the key inhibiting factors in the development of future vaccines are the development timelines. There are indicators that these timelines are prohibitively long (16). In 1996, the development of a human vaccine from preclinical to licensure was estimated to be around 10 years–nowadays it is between 15 and 20 years (16–18). This does not even account for the time it takes to determine and communicate the unmet medical need for the vaccine nor the time it takes to globally deploy it (18).

A direct consequence of the increased vaccine development timelines is the delay in achieving their societal benefit. Additionally, the increasing development costs and a lower market value upon market introduction call for concerns (19). This so-called “productivity gap” thus, causes insufficient market introduction of new products, hampers current research practices, and leads to failure to address unmet medical needs (20–22). Moreover, increasing development timelines threaten the ability to respond quickly to emerging pathogens. These new pathogens emerge at all-time high rates of one per year, and their spread occurs quicker than ever (23–25). Currently the world is facing one such emerging pathogen; SARS-CoV-2 (Covid-19). Considering the immense societal damage it is causing globally, the need for accelerated vaccine development is higher than ever before (26, 27).

Previous scientific papers on vaccine development timelines discuss various opportunities to shorten the vaccine development timelines. Most of these opportunities come in the form of technological advances made over the past two decades in the fields of biomedical sciences, biochemistry, and computational sciences (18, 28–30). These technological advances include rapid isolation of human monoclonal antibodies, synthetic vaccinology, and atomic level proton engineering (31–33). Next to this, as a result of the Ebola, Zika, and SARS outbreaks, steps were taken to better align, organize and regulate vaccine development in times of crisis (34). While these improvements have allowed us to now accelerate Covid-19 vaccine development to truly “pandemic speeds” (35) they are still insufficient considering the currently omnipresent societal damage.

Less has been published on the overall causes for delay in vaccine development. A lack of knowledge on these causes could impede the adoption of the aforementioned technologies, and limit the identification of novel opportunities for improvement (36). Several causes are mentioned by different researchers; including the limitations of current animal models and lack of knowledge concerning the human immune system (23), challenges in the cooperation between academia and industry, and regulatory barriers (18), and challenges specific to emerging pathogens (16).

A limitation of these studies, however, is that by approaching vaccine development as a linear process, these studies fail to account for the complex nature of vaccine development. This can lead to a mechanistic interpretation that is bound to overlook several important factors of innovation (37, 38). Especially during Covid-19, aspects such as, the parallelization of activities is increasingly important (35). The present study therefore aims to identify causes for delay, as experienced by key-opinion-leaders (KOLs) in the vaccine development process and provide context to their cause and impact. To increase the relevance of the findings, an additional goal is to determine whether these identified causes are equally detrimental regarding the Covid-19 vaccine development.

## Methods

This mixed-method study identifies, analyses, and prioritizes innovation barriers as a means to understand deficiencies in vaccine development. Innovation barriers are understood as factors that negatively influence the vaccine innovation process and hamper the commercial use of innovations in the field (39). Only by identifying them and understanding their context can actions be taken to eliminate or mitigate them.

This study featured qualitative and quantitative data collection methods. First, qualitative interviews were conducted with Key-Opinion-Leaders (KOLs) in the field of vaccine development. KOLs were asked to identify barriers and come to an integrated perspective on the important issues delaying the vaccine development process. This information was used to construct a root-cause tree that showed the interrelations of barriers (20, 39–42). The interviews took place between April-June 2019 (before the Covid-19 pandemic). To increase practical relevance of the findings, a larger and more international group of KOLs was approached during the Covid-19 pandemic (June–July 2020) for a quantitative evaluation and prioritization of the identified key barriers. This was done both in the context of general vaccine development and Covid-19 vaccine development.

The Vaccine Innovation Cycle (40) is used as a conceptual model of the vaccine development process to provide context to the identified innovation barriers. The Vaccine Innovation Cycle describes the activities that take place in vaccine innovation. Since many activities in vaccine innovation are performed in parallel, the model consists of multiple, concentric rings, with each ring depicting a separate work stream. Each workstream is further detailed in distinct stages and gates. Some of the activities are clearly defined and carried out in sequential workstreams of relatively predictive order and timing. These workstreams include research and development, GMP production, and market preparation, registration, and introduction. Other activities occur at undefined moments throughout the vaccine innovation process, i.e., activities related to manufacturing, and funding and business development. Finally, a number of workstreams occur continuously during certain phases of innovation, and are defined as monitoring activities, i.e., product monitoring, market monitoring, innovation project monitoring, portfolio monitoring, and public affairs monitoring.

### Participant Selection

KOLs were characterized as influential individuals within the extensive and rapidly changing field of vaccine development. To account for the wide range of activities within the vaccine development process, we aimed to include KOLs with various backgrounds in vaccine development. Accordingly, several identification and recruitment methods were employed: online sources included LinkedIn, patent databases, scientific publications, conference attendance lists, clinical trial dossiers, and organization websites. Lastly, all participants were asked to propose further, potential participants, following the snowball method. To increase the likelihood of participation a personalized invitation letter was send.

Potential participants were considered eligible if their backgrounds clearly demonstrated expertise with the subject matter. Furthermore, only participants with five or more years of experience in vaccine development were included. To account for the need to identify cause and effect of key barriers that span multiple domains of vaccine development, interview participants were required to have knowledge concerning multiple domains of the innovation cycle.

### Interviews

To identify the barriers, a semi-structured interview design was employed. The interviews started with oral informed consent on data collection for this research, respondents were anonymized, and transcripts were not being made publicly available. Interviews were conducted by phone, in person, and using Skype or WebEx. They lasted between 25 and 120 min and were securely recorded using an Android mobile phone and a Roland R-09HR digital recording device.

The interview structure was based on a topic list containing four sets of questions; (1) interviewees experiences on development timelines in the last 10–15 years, (2) identifying steps in the vaccine development process that take a lot of time, (3) possible solutions to the described bottlenecks and (4) confirmation of all perceived barriers and additional comments. During the interviews, the KOLs were asked to identify barriers hampering or slowing down any part in the total vaccine development process. Thereby, we were able to find a broad scope of barriers. In order to maximize the number of identified novel barriers per interview, the conversations were steered away from barriers mentioned in previous interviews. Instead, KOLs were asked to think of “new” barriers not yet mentioned by former interviewees. For each identified barrier exploring questions were asked to establish cause and effect and determine if the KOL could identify any opportunities that could address the barrier. At the end of each interview, as a member check, KOLs were presented with a brief summary of the discussed barriers.

### Data Analysis–Interviews

Interviews were manually transcribed by the interviewer, and uploaded in Atlas.ti for further analysis. The data analysis process was designed to identify barriers and opportunities from the transcripts and provide information on how they were connected to each other. First, transcripts were examined independently by both main authors in an open coding approach. To this end, text fragments were given a short descriptive code that distilled the underlying key barrier. Subsequently, the researchers merged their analyses. Where interpretations differed, a discussion was initiated that resulted in a shared interpretation.

Next, and in cooperation, researchers performed a root-cause analysis, where causal links were identified between the identified key barriers. This resulted in the deduction of key barriers, categorized per theme, and their causal factors. Barriers that were of a more systemic nature were placed as root causes, while more symptomatic barriers were placed close to the key themes. Only when respondents indicated different causal factors contributing to the main barriers were they incorporated in the root cause analysis.

Finally, to be able to discern the impact of the identified key barriers on the vaccine innovation process, the barriers were mapped to the Valorisation Innovation Cycle (VIC) (40), based on an analysis by the two main researchers of the stages that are delayed due to each barrier's impact.

### Questionnaire

To evaluate the impact of the identified key barriers on vaccine development timelines, an online questionnaire was composed using Qualtrics software. The anonymous online questionnaire was piloted and, after finalization, distributed among 846 KOLs. Questionnaire data collection took place from the 8th of June until the 21st of July.

On the introduction page, participants were informed about the purpose of the questionnaire, its length, and the anonymity of the data analysis. They could only proceed by clicking “accept” and thereby giving informed consent. After completion, respondents were provided an option to leave their e-mail address to receive the finalized article and be excluded from reminder emails. These reminders were sent 7 & 14 days after the first invitation. If partially completed, respondents could adapt or finish their response for up to 1 week after their last activity. Following this, or after completion of the questionnaire their data was locked, thereby preventing ballot boxing.

The first questions aimed to determine the demographics of the respondent, as well as, if they were familiar with Covid-19 vaccine development. Thereafter, using a 7-point Likert scale, respondents were asked to indicate for *each* key barrier the impact of that barrier on general vaccine development timelines. If the participant had indicated to be familiar with Covid-19 vaccine development, they were also asked to indicate the impact of each barrier on the Covid-19 vaccine development timelines. Note, that there was an “I don't know” option in each scale. A secondary goal of the Likert scale evaluation was to familiarize the participant with all barriers. This ensured cognitive engagement and allowed for a more reliable response in the second set of questions. There, respondents were asked to list *the three most impactful* barriers on the general and – again, only if familiar–Covid-19 vaccine development timelines.

Finally, two validation questions were asked. The first question invited the respondents to name a barrier that, according to them, is very impactful but rarely experienced, or an existing barrier that is not impactful but often experienced. The second validation question was whether the accumulated barriers were a good reflection of the field. If respondents indicated to (strongly) disagree with that statement, they were asked to name at least one additional barrier they found highly impactful.

### Data Analysis–Questionnaire

Questionnaire results were processed and analyzed using SPSS (version 23.0.00). Descriptive statistics were imported into Microsoft Excel for graphic analysis. For all additional barriers, mentioned by the respondents, it was checked whether they were already present in the root-cause analysis or new.

To prioritize the barriers based on their impact on vaccine development timelines, questionnaire respondents were asked to name and rank the three most impactful barriers during general times. Those that indicated to be familiar with Covid-19 vaccines were also asked to rank the barriers for Covid-19 vaccine development. Each barrier was assigned points depending on the number of times it was listed as the most impactful barrier (3 pts.), second most impactful (2 pts), and third most impactful barrier (1 pt.). The accumulated number of points awarded to each barrier (sum score) divided by the number of points attributed in the situation was used as a measure of relative impact.

The following formula was used to determine relative impact based on the top-3 data:

$$\begin{array}{l} {\%\;of\;total\;sumcore}_{B,S}\\ \;\frac{\sum \left(\left(n_{r1}*3\right)+\;\left(n_{r2}\;*2\right)+\;\left(n_{r3}\;*1\right)\right)*100}{\sum \left(\left(n_{r1}*3\right)+\;\left(n_{r2}\;*2\right)+\;\left(n_{r3}\;*1\right)\right)} \end{array}$$

B, barrier; *n*, number of times; R1/2/3, rank1/2/3; S, Situation (general/Covid-19).

Visual analysis is based on relative sum scores as described above. Since a number of participants had indicated not to be familiar with Covid-19 vaccine development, fewer points were attributed to barriers in the Covid-19 environment than in the general development. This was corrected for by dividing the sum score by the total number of points awarded in the corresponding situation (general or Covid-19).

### Comparing the Average Impact of the Barriers on General Vaccine Development and Covid-19 Vaccine Development

A paired samples *t*-test was conducted to explore the hypothesis whether KOLs rated the key barriers' impact on vaccine development timelines on average differently regarding situational characteristics (non-pandemic vs. Covid-19). To test this hypothesis, mean scores of the Likert-scale impact-ratings across all barriers for each of the two situations were computed and tested against each other in a within-subject design. As the choice of analysis for Likert-scale data is consistently in discussion we also conducted non-parametric tests to investigate our findings more closely.

## Results

### Interview Demographics

Twenty one KOLs of 245 (9%) invited KOLs agreed to participate in the interviews. After reconsidering their backgrounds, one of the KOLs was removed from the group of participants. All areas of work were represented by the interviewees, with 5 working at a CRO (25%), seven in industry (35%), three in government/regulatory (15%) and five in academia (25%). All interview participants had worked in the field of vaccine development for over 5 years, and all but one over 10. Geographically, the group was not well-representative, with all respondents living in either the United States (20 %) or Europe (80%). An overview of participant demographics is provided in Supplementary Table 1.

### Data Saturation and Root Cause Analysis

A root cause analysis and de-duplication efforts resulted in the identification of 20 key barriers grouped in seven larger themes. The 20 key barriers are causally linked/explained by 35 causal factors and 58 root-causes. Five root-causes were found to be linked to more than one key barrier. The results are presented following seven themes. The aim of this qualitative analysis was to identify which barriers occur, and what their underlying patterns are. Specifically, we strived to reach saturation of key barriers. Saturation occurs when several sequential interviews reveal no new key barrier. In interviews 13 through 20 only a single new key barrier was identified (interview 18, see Supplementary Figure 1). Based on this, we concluded to have reached data saturation.

### Vaccine Candidates and Technologies Get Stuck in Discovery and Realization Phases

**Figure 1:**
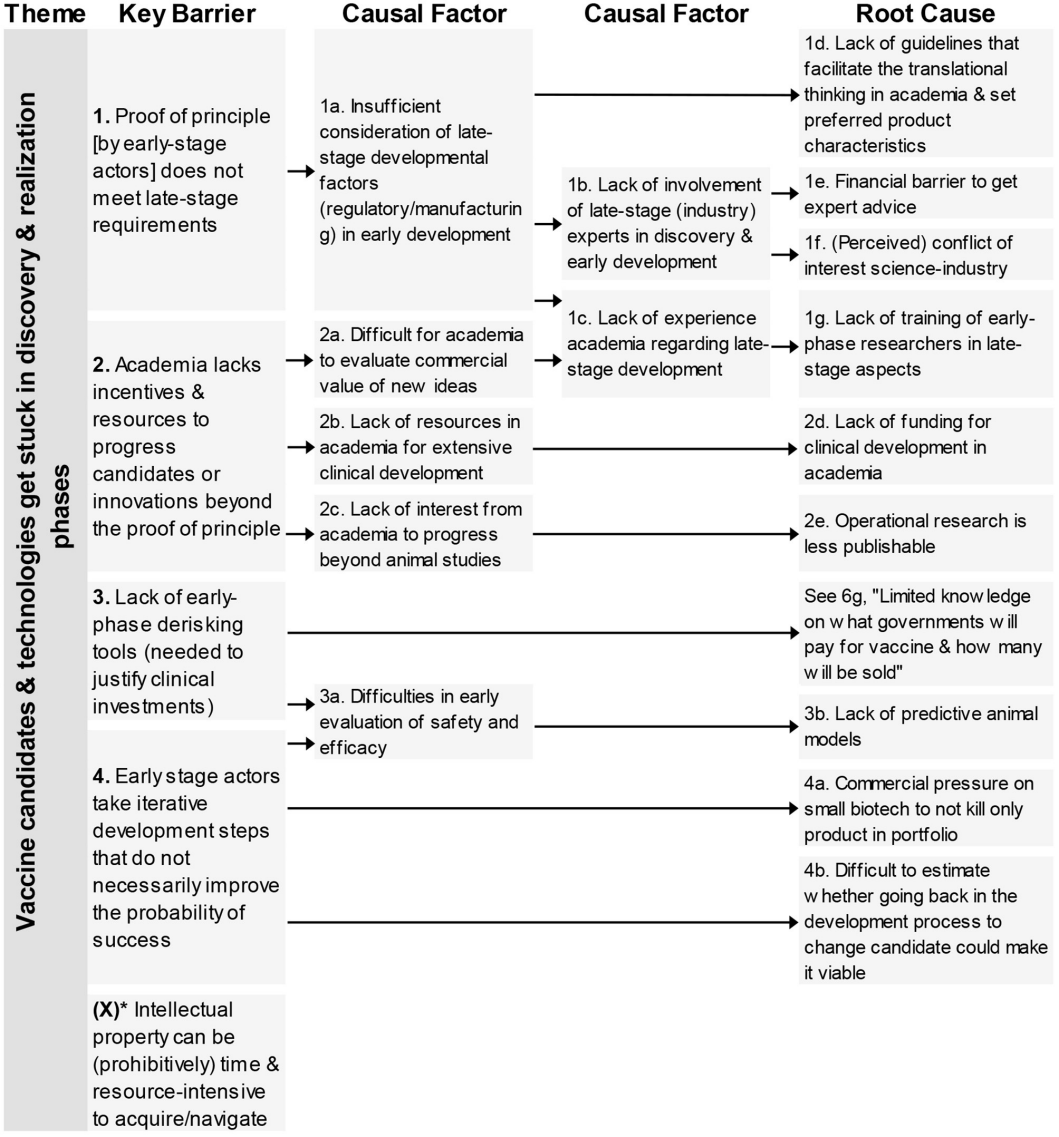
Root-cause analysis of the four key barriers classified under the theme “Vaccine candidates & technologies get stuck in discovery & realization phase.” Columns are causally structured. (Left) key barriers are listed that are caused by causal factors (middle), which in turn find their origin in root causes (right). *Key Barrier X was added based on questionnaire responses.

This first theme contains five key barriers (Figure 1) that impact the transition from the discovery & realization phase to the (late stage) clinical trials. Four of the barriers were identified in interviews, one in the questionnaire. The key barriers deal with lacking proof of principle, academia not developing vaccines far enough, unnecessary development steps and prohibitive intellectual property.

In total, nine root-causes and six causal factors were identified. The causal factors deal with limitations in the consideration of late-stage factors, lack of resources or interest. The root-causes underlying these barriers related to different aspects; lack of guidelines, lack of funding, lacking cooperation between academia-industry, concerns on possibilities to publish and lack of predictive animal models. One root-cause also impacted a barrier from a different theme (see 6g).

### Lack of Investments by Private Companies

**Figure 2:**
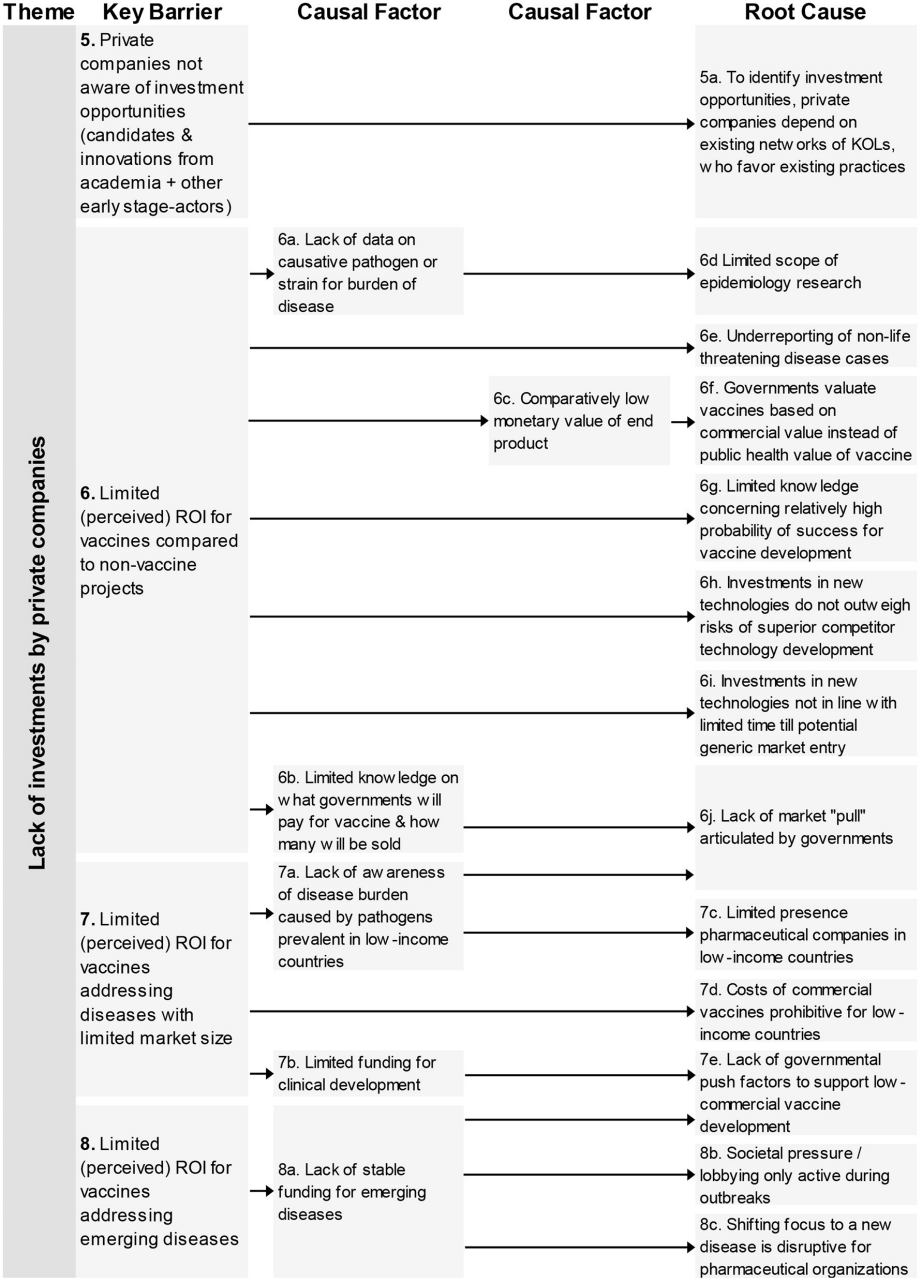
Root-cause analysis of the four barriers classified under the theme “Lack of investments by private companies.” Columns are causally structured. (Left) key barriers are listed that are caused by causal factors (middle), which in turn find their origin in root causes (right).

The four key barriers (Figure 2) in this theme refer to the unawareness of private companies regarding vaccine candidates generated by academia, and three barriers that impact the (perceived) ROI of a vaccine candidate, the comparison with non-vaccine products, limited market size, and region of impact of pathogen.

For these barriers 13 root-causes and six causal factors were mentioned. The root-causes contain the following aspects: limited scouting practices by industry, limited epidemiology research, limitations on the impact and return of investments, and lacking governmental push and pool factors.

### Fundamental Barriers on Pathogens and Immune System

**Figure 3:**
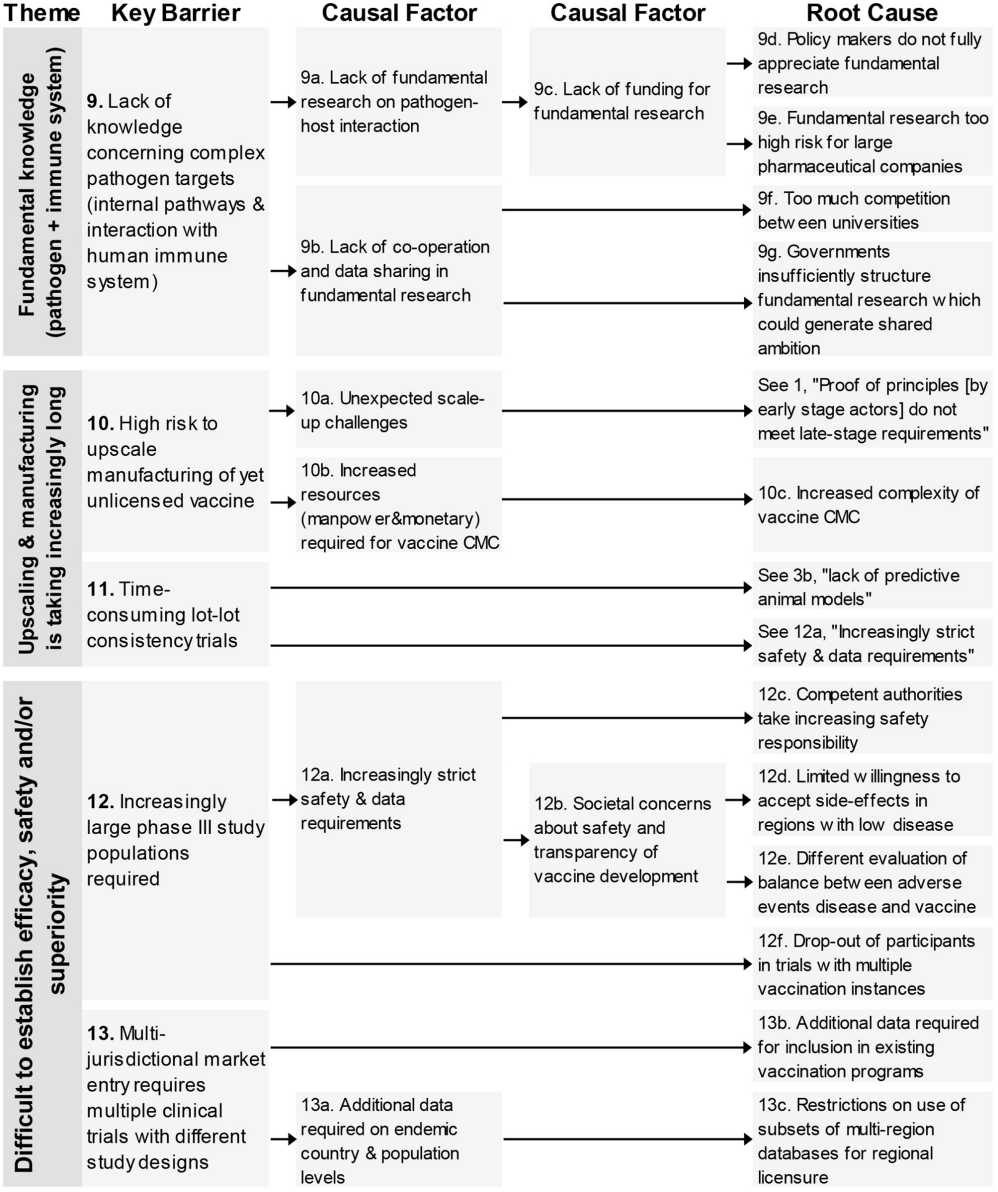
Root-cause analysis of the three barrier themes classified under the themes “Fundamental Knowledge on pathogen and immune system,” “Upscaling & manufacturing is taking increasingly long” and “Difficult to establish efficacy, safety, and/or superiority” (Part 1). Columns are causally structured. (Left) key barriers are listed that are caused by causal factors (middle), which in turn find their origin in root causes (right).

One key barrier–“lack of knowledge concerning complex pathogen targets”–is related to this theme (Figure 3). The causal tree revealed four different root-causes relating to the way policy makers, governments, and large pharmaceutical companies consider fundamental research and the lack of cooperation amongst academia in their (fundamental) research efforts.

### Upscaling and Manufacturing Is Taking Increasingly Long

The two key barriers (Figure 3) in this theme were related to the high risk of upscaling manufacturing of yet unlicensed vaccines and time-consuming lot-lot consistency trials. One of four root-causes was unique concerning the increased complexity of vaccine CMC, the other three root-causes originated from other barrier themes (see 1, 3b, 12a).

### Difficult to Establish Efficacy, Safety, and/or Superiority

**Figure 4:**
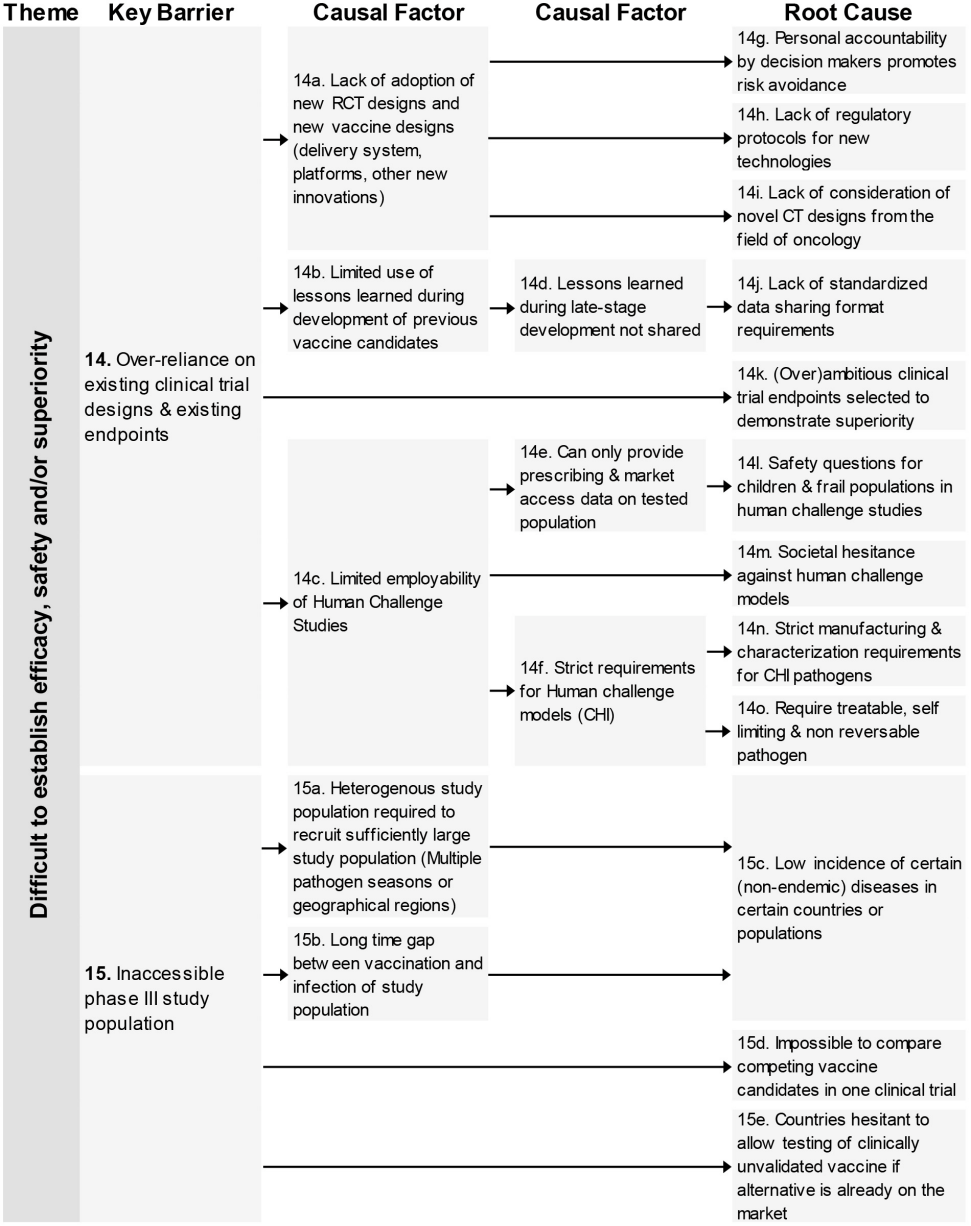
Root-cause analysis (part 2) of the theme “Difficult to establish efficacy, safety, and/or superiority.” Columns are causally structured. (Left) key barriers are listed that are caused by causal factors (middle), which in turn find their origin in root causes (right).

This theme consists of four key barriers (12–15) relating to different clinical trials aspects; the large study populations, the difficulty to test in the required population, an over-reliance on existing designs and the need for multiple clinical trials with different designs (see Figures 3, 4).

In total 18 root-causes and 11 causal factors were identified for these four key barriers. The root-causes covered many aspects including an increased safety requirement by competent authorities, lack of consideration of CT designs from oncology, reasons for not adapting novel innovations and low incidence of certain diseases.

Four key barriers in the theme “Long time between trials” are related to the time it takes to make organizational decisions, develop clinical trial protocols and dossiers, analyse clinical trial data, and having it reviewed by regulatory authorities.

In total nine root-causes and five causal factors were identified. The root-causes underlying these barriers related to complexity on several different levels concerning, increasing number of stakeholders, allocation of budget, turnover of experienced personnel, increasing number of datasets, and country-specific regulations.

### Market Inefficiencies

**Figure 5:**
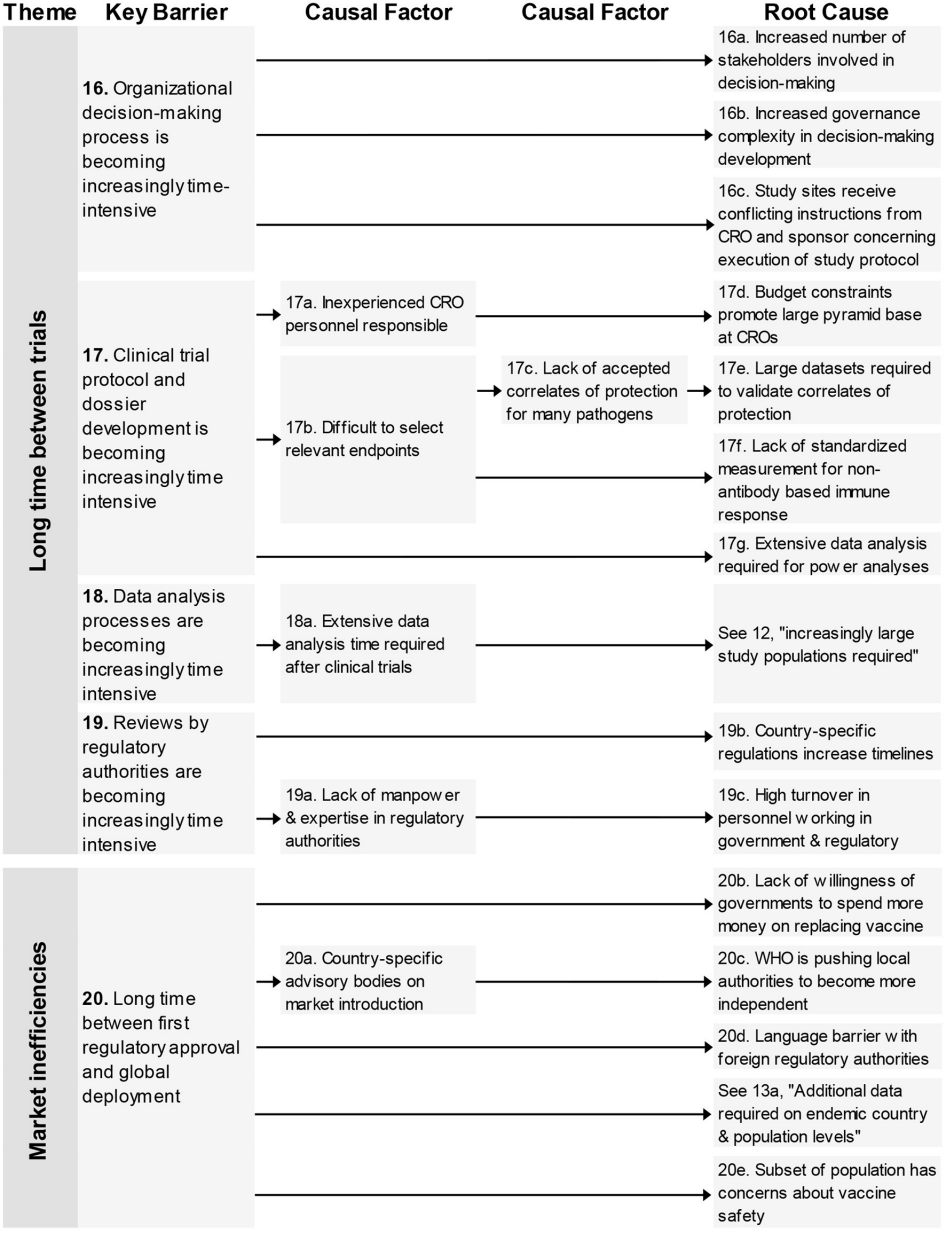
Root-cause analysis of the themes “Long time between trials” and “Market inefficiencies.” Columns are causally structured. (Left) key barriers are listed that are caused by causal factors (middle), which in turn find their origin in root causes (right). Long time between trials (Figure 5).

This last theme contains one key barrier; “long time between first regulatory approval and global deployment” and finds four unique root-causes concerning governmental investments, regulations from the WHO, language barriers for authorities in the different countries, and the concerns from a subset of society on vaccines (Figure 5).

### Barriers Plotted in the VIC-Stage-Gate Model

**Figure 6:**
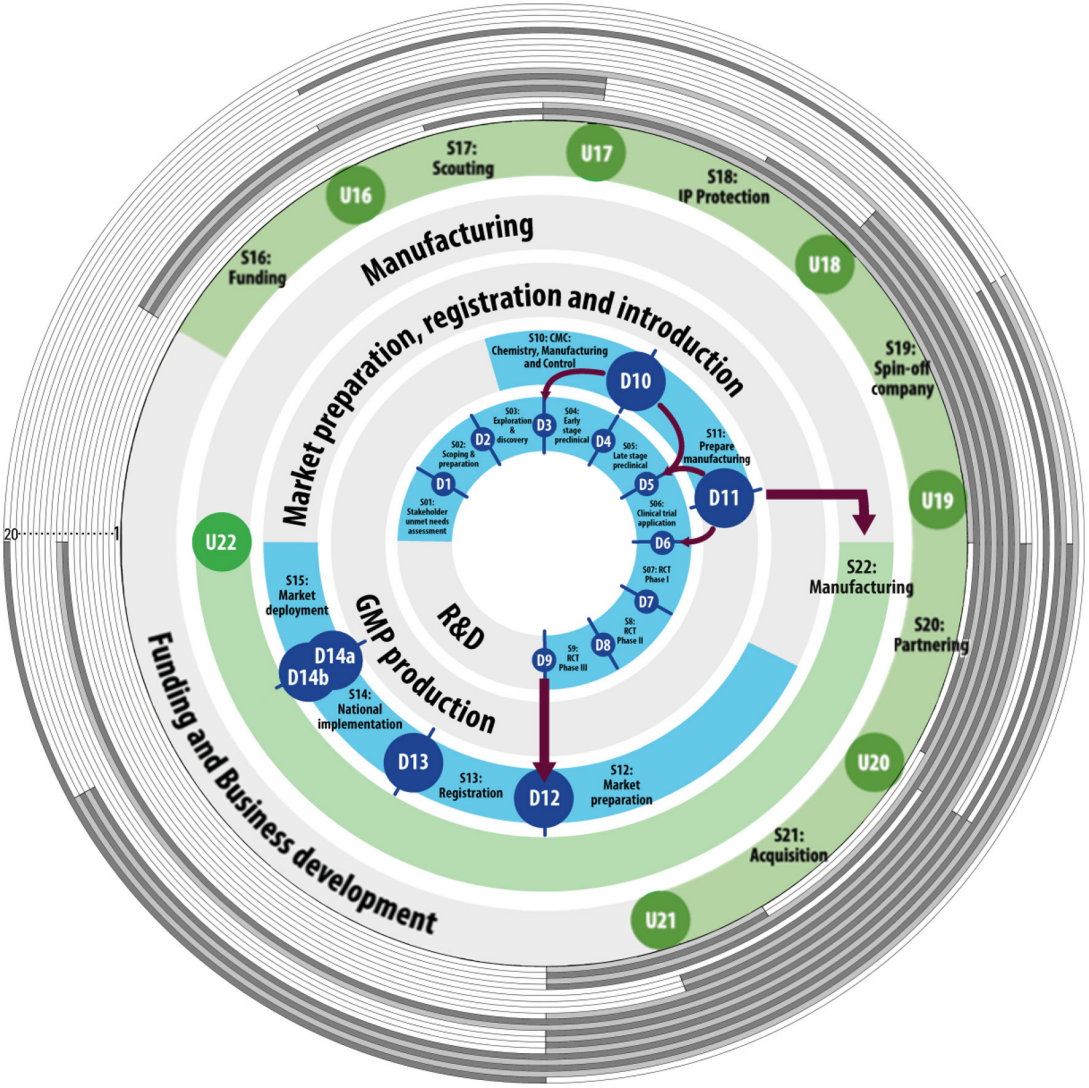
Barriers have their impact across the vaccine innovation cycle. The inner blue and green circles represent the defined and undefined stage-gates of the Vaccine Innovation Cycle [adapted from (40)]. The monitoring stage-gates are left out for readability purposes. Numbered from 1 to 20, the gray colored circles each represent one of the 20 identified key barriers. Where the circle is shaded, the barrier impacts the corresponding stage in vaccine development.

All 20 identified key barriers were mapped to the VIC to illustrate their location of impact in the vaccine development process (see Figure 6). The figure shows that all phases of vaccine development are impacted by the key barriers identified in this study. The area that is impacted by most barriers starts at late stage preclinical and ends at the phase 3 clinical trials This area is impacted by all but one barrier.

### Questionnaire Demographics

A total of 131 respondents of 864 invited KOLs responded to the survey, 39 responses were excluded based on finishing <75% of the survey (*n* = 31), or not indicating to have worked within the field of vaccines for at least 5 years (*n* = 8). Leading to a final sample of *N* = 92 (out of 864, 11%) responses by qualified KOLs. The largest group of respondents indicated to currently live in Europe (47%), followed by North-America (33%), and Asia (11%). Also, experts from Australia (*n* = 1), Africa (*n* = 5), and South-America (*n* = 2) responded to the survey. The majority of the participants (*n* = 72, 81 %) indicated to be familiar with the Covid-19 vaccine development, 17 participants were not. An overview of participant demographics is provided in Supplementary Table 1.

Our sample can clearly be divided into three major groupings of regulation agencies participants mostly interact with: 23 % named the EMA, and 26 % the FDA, while 37% of responses represent a very diverse field of other regulatory agencies. Notably, 14% of participants did not respond to this question. The responding KOLs represent all major vaccine fields, with 64% indicating to currently be working on bacterial vaccines, 86% on viral vaccines, 25% with parasitic vaccines, and 15% with cancer vaccines. Numerous KOLs indicated to work on multiple vaccine types.

Lastly, each included work area of participating experts (the stakeholder group it belongs to) was represented in the questionnaire. Similarly, participants were associated with diverse and evenly distributed organizations, including academia (27%), government or regulatory agencies (22%), industry (26%), as well as Non-profits or NGOs (25%). Only two participants indicated to relate their work mostly with Contract research organization (CRO) which led to the inclusion of this theme within “industry.”

Eighty percent of the participants agreed with the statement that “The accumulated barriers represent the field well; I do not have any to add.” Those that did not agree to the statement mentioned several barriers. All but two of these barriers were already mentioned in the interviews, and present as causal factors or root-causes in the RCA. Two respondents mentioned a new barrier that was not yet present in the RCA concerning intellectual property (IP). The barrier was added to the RCA as a new key barrier in the theme; “Vaccine candidates & technologies get stuck in discovery & realization phases (see ^*^in Figure 1).”

### Perceived Difference of the Barriers' Impact on General- and Covid-19 Vaccine Development Timelines

Based on the Likert data, we tested the hypothesis that the 20 identified key barriers are less impactful on general vaccine development timelines than on Covid-19 vaccine development timelines. A paired samples *t*-test supported this hypothesis (*M*_*g*_ *(SD)* = 5,58 (0, 64); *M*_*c*_ *(SD)* = 4,40 (1, 2); t (61) = 8,68; *p* < 0.000). Non-parametric tests were in accordance.

### Prioritization of Barriers on General- and Covid-19 Vaccine Development Timelines

All 20 key barriers identified for general vaccine development were included at least once in the top 3's of most impactful barriers according to the KOLs. The fact that each barrier was considered by a KOL to be one of three most impactful can be considered a validation of their inclusion in the RCA. This was also the case for all but two barriers regarding Covid-19 vaccine development. See Supplementary Figure 2.

For general vaccine development, KOLs identified ROI related barriers are of highest impact. Followed by barriers “Academia does progress beyond proof of principle,” “Proof of principle does not meet late-stage requirements” and “Lack of knowledge concerning pathogen target.” However, the distribution of points between all barriers was quite spread out and as reported, none of the barriers was left unmentioned. In contrast to this, the spread of impact sum scores for Covid-19 vaccine development are much more centered around only few barriers. The barriers “Lack of knowledge concerning pathogen target,” “High risk to upscale unlicensed vaccine” and “Proof of principle does not meet late-stage requirements” were deemed of much higher impact than the rest.

When comparting the impact of specific barriers during general vaccine development and Covid-19 vaccine development, large differences appear. The two most impactful barriers during general vaccine development, that relate to a limited ROI, were barely mentioned as one of three most impactful during Covid-19. Other notable differences between the two situations, besides the three most impactful barriers on Covid-19, are “lengthy regulator review durations,” “lack of early phase de risking tools” and “inaccessible phase III study population” See Supplementary Figure 2.

**Figure 7:**
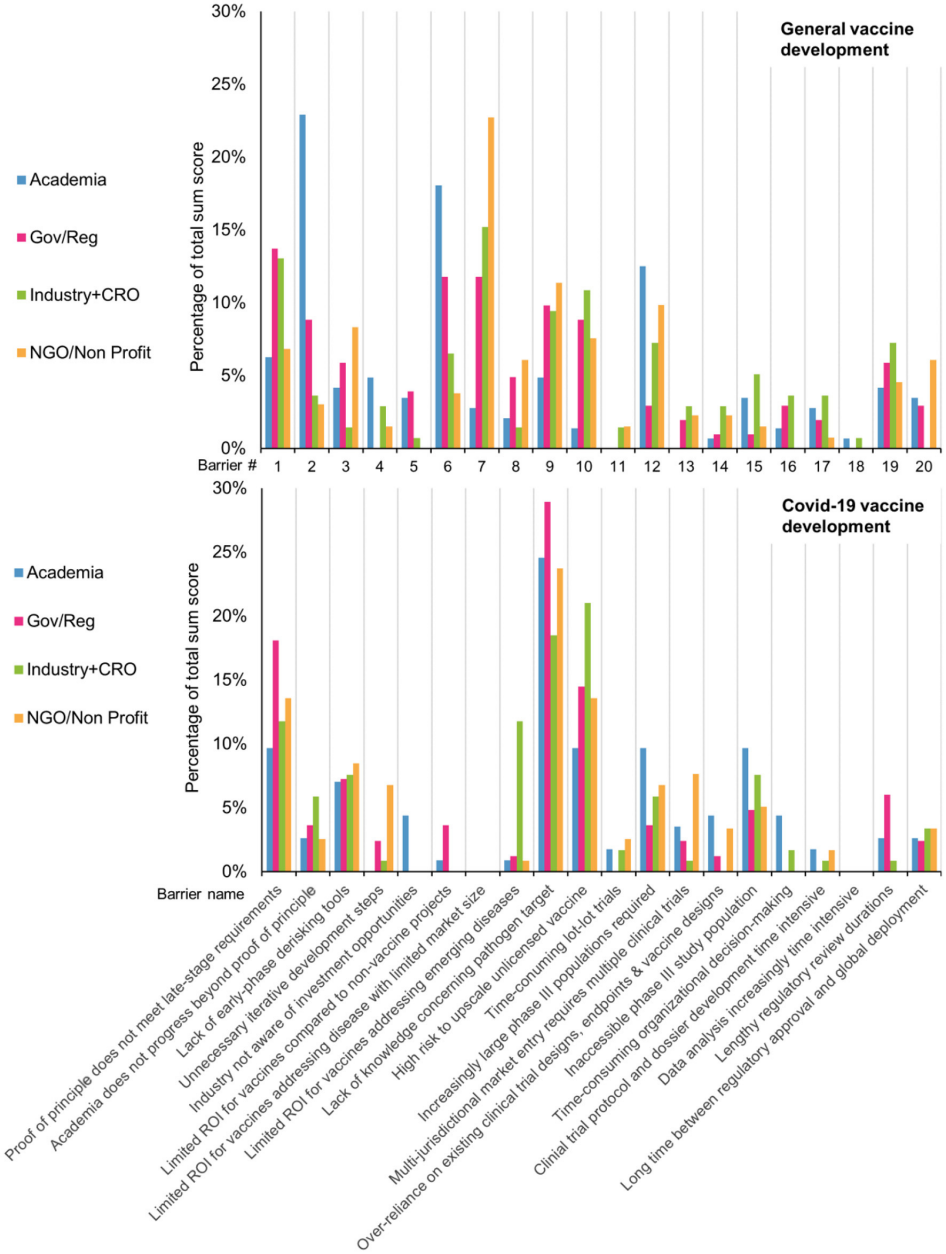
During general development, KOLs from different work areas ranked the impact of each barrier on development timelines differently. In contrast, during Covid-19, the different groups largely agreed on the impact of each barrier.

Furthermore, we investigated whether the rank sum score distribution would differ between the work area (stakeholder group) of the KOLs (Figure 7). That is, whether we would see differences between the stakeholder groups regarding which barriers they deem most impactful. For the general vaccine development, there was some agreement between the different stakeholder groups. Notable is the barrier “Academia does not progress beyond proof of principle,” which was judged the most impactful barrier by KOLs from academia but received far fewer points by KOLs from other stakeholder groups. This indicates that it is considered a major barrier by academia, but not by industry. For Covid-19 vaccine development, there is a large similarity in the impact attributed to each barrier by KOLs from different stakeholder groups. A notable exception is the barrier “Limited ROI for vaccines addressing EM diseases” which industry KOLs considered the second most impactful barrier, while the other KOLs rarely assigned it a top-3 spot.

## Discussion and Conclusion

Here we elucidate the key barriers that delay vaccine development timelines. We show a clear difference in KOL perception of barriers hampering timelines in general vaccine development vs. barriers specific for Covid-19 vaccine development. Furthermore, we show that stakeholder perceptions differ on the most impactful barriers, potentially further hampering effective collaboration.

The most important barriers for general vaccine development relate to their limited ROI, either in small market sizes, or in comparison to non-vaccine projects. In addition, although academia is working on vaccines, they are generally not able to progress their candidates beyond the proof of principle. The root cause analysis shows that these barriers originate from a lack of awareness of disease burden, and limited ROI and funding in low-income countries. The root causes mentioned relating to the limited ROI for vaccine development compared to non-vaccine projects suggest an underestimation of general public health, limited knowledge concerning probability of successful vaccine development, and hesitancy concerning investments in new technologies. These causal factors fit previous findings showing that increased resource mobilization efforts over the years have resulted in more countries introducing new vaccines while optimizing coverage for traditional vaccines (43).

In contrast, only the government stakeholder group, considered this barrier to be (marginally) important during the Covid-19 pandemic.

These findings demonstrate the important catalyzing effect of large funding programs being made available by organizations like GAVI and CEPI to further vaccine development (44–46). Rethinking the mechanisms to engage governmental and NGO funding also during peace time, can substantially increase our preparedness for emerging infectious diseases, and address large unmet needs in currently neglected infectious diseases (47, 48). In addition, to realize a positive ROI on investment for regular immunization programs for limited market size, it is essential that governments and donors provide the requisite investments (20, 49). During regular vaccine development, especially the NGO and industry stakeholder groups regard this barrier as highly impactful, suggesting awareness of unmet medical needs but a lack of capital and intellectual investments. Attributing high priority to this barrier of limited ROI in small market sizes, is in line with previous findings that the market for low-income countries is not attractive for industry (50). During the Covid pandemic none of the stakeholder groups found this barrier being of impact.

A second, highly prioritized barrier during general vaccine development relates to academia not progressing their candidates beyond proof of principle. A broad variety of causal factors were identified that cause this poor hand-off point between academia and industry, some of which have already been described before (51–53). Also, knowledge by academic researchers on how to conform to late-stage demands is lacking and is usually acquired from external sources (54). Interestingly, during general vaccine development academic KOLs judged this barrier much more impactful than during covid-19. This suggests that during war time much more willingness exists by industry to contribute to early-stage development and progress these candidates through the vaccine innovation cycle.

To ensure such collaboration continues also during peace time, the right combination of stakeholder type and outcome measure of interest for each stakeholder group is needed (55). During the interviews KOLs support arguments for incorporation of late-stage knowledge through public private partnerships or Max-Planck-like centers of applied research (48). They also strongly advised to open communication channels with regulatory agencies as early as possible. Both to gain insight into the regulatory agencies' perspective, but also to see if the regulatory demands can be adapted to the specific context. Interestingly, both during general vaccine development and Covid-19 vaccine development government and industry stakeholders rated the lack of progression by academia beyond the proof of principle as the most impactful barrier, while respondents from the academia stakeholder group considered this barrier to have limited impact. This suggests that the ambitions of academic stakeholders are not in line with the expectations and roles as seen by government and industry (56).

During regular vaccine development all stakeholder groups attributed moderate importance to a lack of knowledge concerning complex pathogen targets. This barrier was much more pressing during the Covid-19 pandemic, where it is seen by all stakeholder groups as the most impactful barrier. Interestingly, this discrepancy between a consideration that “all is known” about existing pathogens, while simultaneously a lack of fundamental insight limits progress was already described before for rabies (57). Considering this barrier is being perceived as most impactful during the development of a human Covid-19 vaccine shows that no lessons are learned (yet) from the development of veterinary coronavirus vaccines in a “One Health” context (58). This not only reinforces the argument for continuous investments in fundamental research to improve preparedness, but also calls for improved collaborations across human and veterinary disciplines (59).

The second most impactful barrier during Covid-19 vaccine development relates to the high risks associated with upscaling and manufacturing yet unlicensed vaccine candidates. It was deemed especially impactful by industry KOLs. Despite a clear recognition of this barrier in the pandemic situation, J&J and others started early in the vaccine development with the production of different vaccine candidates that were at that point still early-stage (60, 61). Herein they were aided by NGOs/non-profits and governments who also assigned high impact to the barrier (44). In general vaccine development, while in the top 3 for industry, academia judged the barrier to be one of the least impactful. This divide reinforces the notion that academia is unfamiliar with the needs of late stage vaccine development.

One last barrier mentioned having a high impact on Covid-19 vaccine development is the barrier concerning the proof of principle (by early-stage actors) not meeting late-stage requirements. This lack of early conformation is a product of the highly complex nature of vaccine development that incorporates far-removed stakeholders with distinct needs. When clinical-stage needs are not met early, it results in vaccines that cannot progress beyond the proof of principle. When market needs are not met, it leads to situations like the first mRNA vaccines, whose cold-chain requirements proved difficult even for high-income countries to manage. As such, this barrier is closely connected to several other important barriers in vaccine development.

The current results should be interpreted in light of a view limitations. First of all, key opinion leaders were not geographically distributed. Most respondents were from the global North, leaving out perspectives from the global South. As a result of this sampling, innovation barriers for instigating, or expanding vaccine development in the global South are not included in this study. Second, vaccine development was broadly conceptualized. This enabled the connection of themes and barriers across phases and areas of vaccine development. Simultaneously, although KOLs were sampled broadly from across key relevant areas in the process, notable stakeholders that were not included were patients, healthcare personnel, and non-governmental organizations.

In conclusion, the current study demonstrates that barriers hampering timelines in vaccine development are present across the Vaccine Innovation Cycle, with the phase between late development and end of phase III clinical trials being impacted by most barriers. Quantitative analysis shows that stakeholder perceptions on the impact of barriers differ, which could further delay effective collaboration and hence vaccine development. Prioritizing the impact of barriers in general and Covid-19 vaccine development, shows clear differences that can be used to inform policies to speed up development in both pandemic and non-pandemic situations. These new findings should be put in perspective of both access & benefit sharing under the Nagoya protocol (62) and technological solutions relating to ownership and inventorship under Covid-19 (27).

## Data Availability Statement

The raw data supporting the conclusions of this article will be made available by the authors, without undue reservation.

## Author Contributions

EC and PH are both professors of the two research groups for which this research was conducted. LB is promotor and ultimately responsible. All authors contributed to the article and approved the submitted version.

## Conflict of Interest

The authors declare that the research was conducted in the absence of any commercial or financial relationships that could be construed as a potential conflict of interest.
