# Supplementary material for: Barriers Influencing Vaccine Development Timelines, Identification, Causal Analysis, and Prioritization of Key Barriers by KOLs in General and Covid-19 Vaccine R&D
Source: Front Public Health. 2021 Apr 20;9:612541. doi: 10.3389/fpubh.2021.612541 (PMC8096063; doi:10.3389/fpubh.2021.612541)
Supplement: Supplementary file 1 [file Data_Sheet_1.PDF]

Supplementary Material

1 Supplementary Data

Supplementary Material should be uploaded separately on submission. Please include any supplementary data, figures and/or tables. All supplementary files are deposited to FigShare for permanent storage and receive a DOI.

Supplementary material is not typeset so please ensure that all information is clearly presented, the appropriate caption is included in the file and not in the manuscript, and that the style conforms to the rest of the article. To avoid discrepancies between the published article and the supplementary material, please do not add the title, author list, affiliations or correspondence in the supplementary files.

2 Supplementary Figures and Tables

Supplementary Table 1. Study demographics

		Interviews	Survey
Number of invites sent		250	864
Number of responses		21	131
Number that met criteria		20	75
Continent of residence	Europe	16 (80%)	34 (45%)
	North-America	4 (20%)	28 (37%)
	Asia	-	7 (9%)
	Africa	-	4 (5%)
	Australia	-	1 (1%)
	South-America	-	1 (1%)
Most interacted with regulatory agency	FDA	n/a	22 (34%)
	EMA	n/a	17 (26%)
	MHRA	n/a	5 (8%)
	Other	n/a	21 (36%)
Work relates to: (multiple conditions possible per participant)	Viral	n/a	65 (49%)
	Bacterial	n/a	47 (35%)
	Parasitic	n/a	21 (16%)
Sector of occupancy	Academia	5 (25%)	20 (27%)
	Government/Regulatory	3 (15%) *	19 (25%)
	Non-profit/NGO		19 (25%)
	Industry	7 (35%)	15 (20%)
	CRO	5 (25%)	2 (3%)

* in the interviews, the demographics 'Government/Regulatory' and 'Non-profit/NGO' were grouped

2.1 Supplementary Figures

Supplementary Figure 1. The figure legends are required to have the same font as the main text, 12 point normal Times New Roman, single spaced. Please use a single paragraph for each legend and prepare the figures keeping in mind the PDF layout.

Supplementary Figure 1.

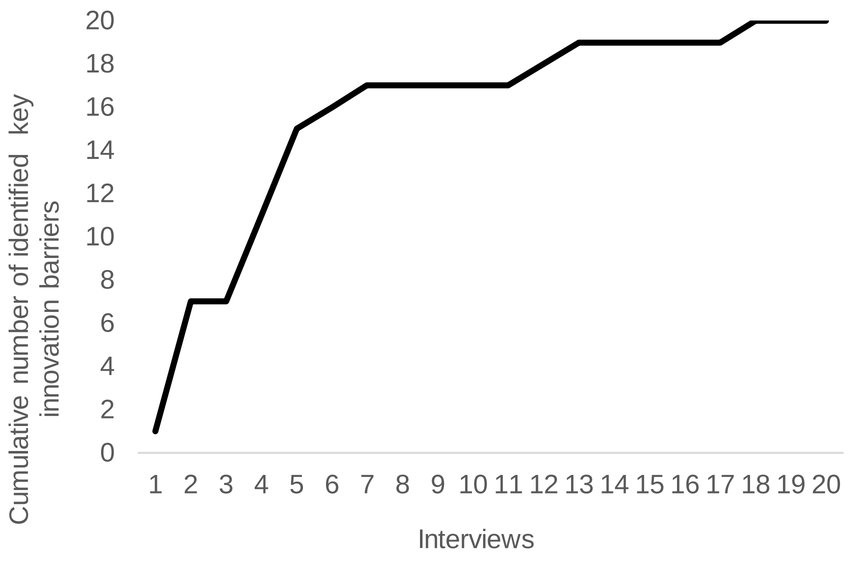

Supplementary Figure 1.
Saturation occurred when several sequential interviews reveal no new key barrier. In interviews 13 through 20 only a single new key barrier was identified.

Supplementary Figure 2.

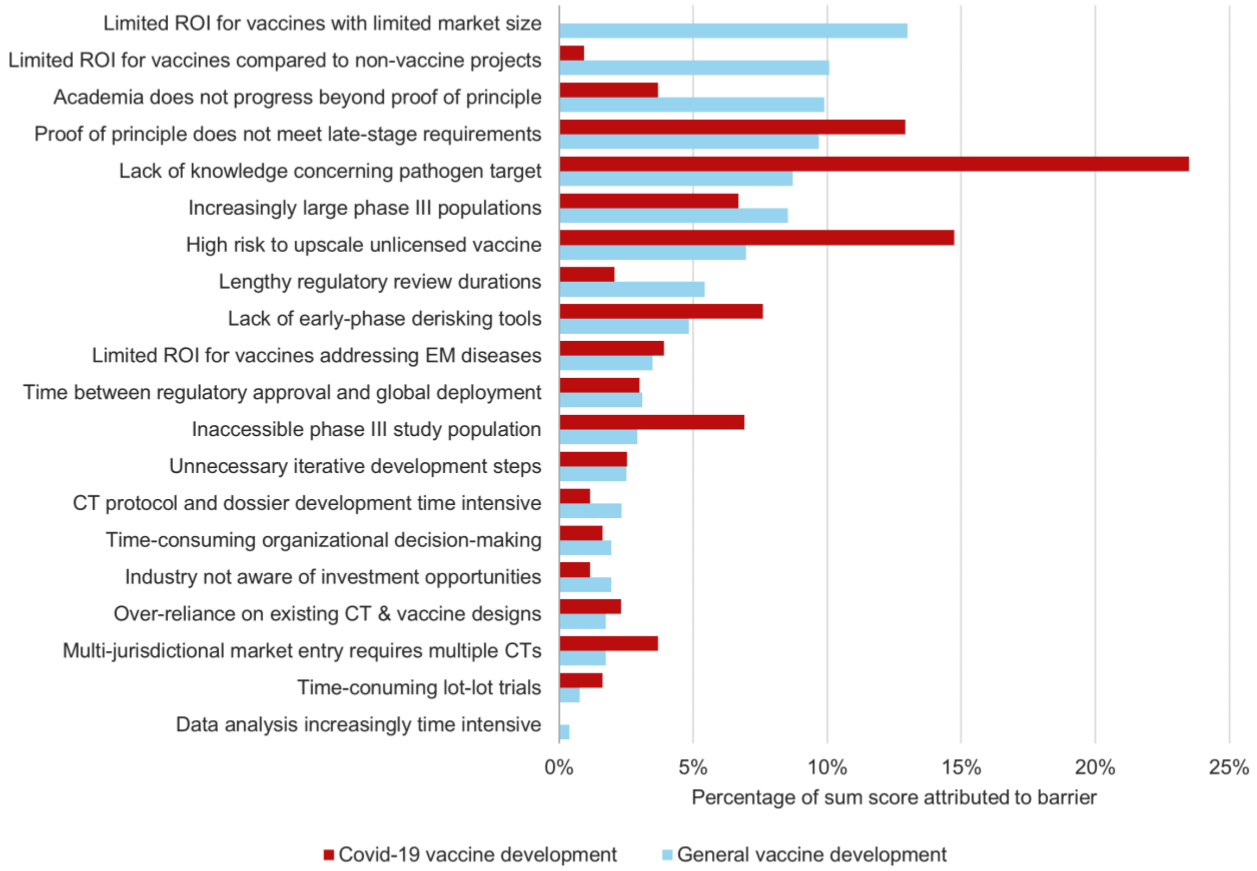

Supplementary Figure 2. Prioritization of the key barriers based on the top 3 ranking, shows a clear difference in priority for the two different situations. For the general vaccine development situation ROI related barriers are perceived to be of highest impact. For the Covid-19 situation one barrier is clearly perceived as highest impact on vaccine development and relates to the lack of knowledge concerning the pathogen target. Barriers in the figure are sorted based on impact on general vaccine development.
